# Supplementary material for: The causal relationship between the human gut microbiota and pyogenic arthritis: a Mendelian randomization study
Source: Front Cell Infect Microbiol. 2024 Nov 26;14:1452480. doi: 10.3389/fcimb.2024.1452480 (PMC11629706; doi:10.3389/fcimb.2024.1452480)
Supplement: Supplementary file 1 [file DataSheet1.zip › mendelian test/ebi-a-GCST90027663.csv_forest.pdf]

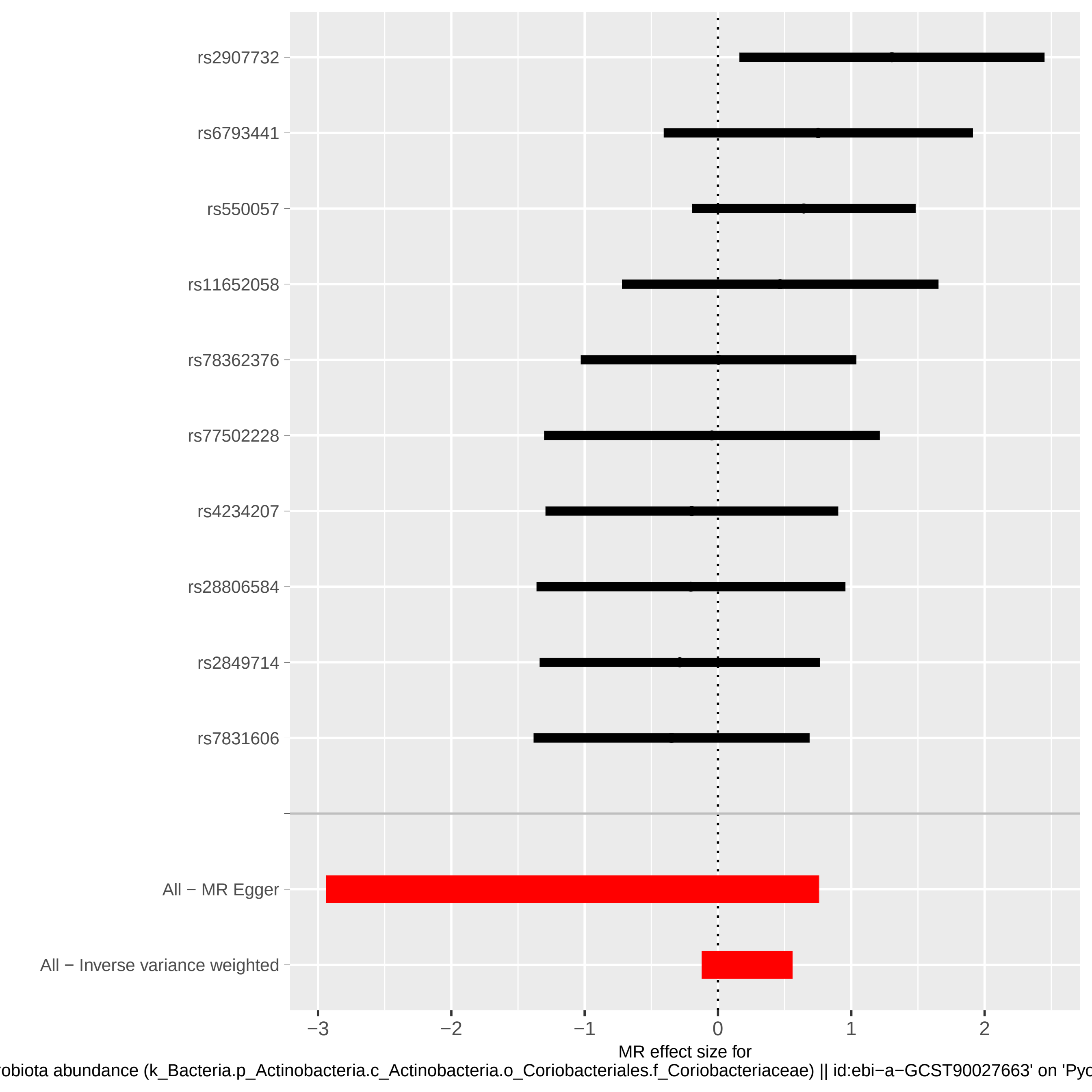
rs2907732
rs6793441
rs550057
rs11652058
rs78362376
rs77502228
rs4234207
rs28806584
rs2849714
rs7831606
All − MR Egger
All − Inverse variance weighted
−3
−2
−1
0
1
2
MR effect size for
robiota abundance (k_Bacteria.p_Actinobacteria.c_Actinobacteria.o_Coriobacteriales.f_Coriobacteriaceae) || id:ebi−a−GCST90027663' on 'Pyc
